# Supplementary material for: Virtual reality as a strategy for intra-operatory anxiolysis and pharmacological sparing in patients undergoing breast surgeries: The V-RAPS randomized controlled trial protocol
Source: PLoS One. 2025 Jul 7;20(7):e0327555. doi: 10.1371/journal.pone.0327555 (PMC12233254; doi:10.1371/journal.pone.0327555)
Supplement: S1 Appendix — (DOCX) [file pone.0327555.s001.docx]

**Virtual reality as a strategy for intra-operatory anxiolysis and pharmacological sparing in patients undergoing breast surgeries: the V-RAPS randomized controlled trial protocol**

**Appendices**

**Appendix A** Model consent from given to participants (only distributed in French)

Pages 2 – 8.

**Appendix B** World Health Organization trial registration dataset

Pages 9 – 10.

**Appendix A** Model consent from given to participants (only distributed in French)

**FORMULAIRE D’INFORMATION ET DE CONSENTEMENT**

| **Titre du projet de recherche :** | Utilisation de la réalité virtuelle comme stratégie d'anxiolyse et d’épargne pharmacologique peropératoire chez les patientes subissant des chirurgies mammaires : l'essai clinique randomisé V-RAPS | | |
| --- | --- | --- | --- |
| **Chercheur responsable du projet de recherche :** | Pascal Laferrière-Langlois, MD, MSc, Professeur Adjoint de Clinique,  Hôpital Maisonneuve-Rosemont, CIUSSS de l’Est de l’Ile de Montréal | | |
| **Co-chercheur(s)/site(s) :** | Joe Zako, Département d’Anesthésiologie et de Médecine de la Douleur, Université de Montréal  Nicolas Daccache, Département d’Anesthésiologie et de Médecine de la Douleur, Université de Montréal  Julien Burey, MD, Anesthésiologiste fellow, Département d’Anesthésiologie et de Médecine de la Douleur, Université de Montréal  Louis Morisson, MD, MSc, PhDc, Anesthésiologiste, Professeur Adjoint de Clinique, Hôpital Maisonneuve-Rosemont  Olivier Verdonck MD, MSc, Anesthésiologiste, Hôpital Maisonneuve-Rosemont, CIUSSS de l’Est de l’Ile de Montréal | | |
| **Membre du personnel de recherche :** | Nadia Godin, RN, Coordinatrice de recherche, Hôpital Maisonneuve-Rosemont  Moulay Idrissi, BEng, MSc, Assistant de recherche, Hôpital Maisonneuve-Rosemont | | |
| **Installation(s) ou site(s) :** | | CIUSSS-CEMTL, Installation Maisonneuve-Rosemont |  |
|  |  |  |  |

**INTRODUCTION**

Nous vous invitons à participer à un projet de recherche parce que vous allez bientôt avoir une chirurgie. Cependant, avant d’accepter de participer à ce projet et de signer ce formulaire d’information et de consentement, veuillez prendre le temps de lire, de comprendre et de considérer attentivement les renseignements qui suivent.

Ce formulaire peut contenir des mots que vous ne comprenez pas. Nous vous invitons à poser toutes les questions que vous jugerez utiles au chercheur responsable de ce projet ou à un membre de son personnel de recherche et à leur demander de vous expliquer tout mot ou renseignement qui n’est pas clair.

**NATURE ET OBJECTIFS DU PROJET DE RECHERCHE**

Depuis quelques années, la réalité virtuelle se développe dans différents domaines de la santé. Cette technologie peut être utilisée en salle d'opération pour offrir une immersion optimale en toute sécurité, sans nuire à la chirurgie en cours.

Les recherches ont principalement porté sur l'utilisation de la réalité virtuelle chez les enfants pour réduire leur anxiété et améliorer leur confort lors de soins potentiellement douloureux, comme les soins dentaires. Chez les adultes, la réalité virtuelle permet également de diminuer l'inconfort et le stress pendant des opérations chirurgicales réalisées sous anesthésie régionale, où les patients sont éveillés et seule la zone opérée est anesthésiée.

Il existe encore très peu d'études sur l'impact de la réalité virtuelle sur les besoins en médicaments sédatifs chez les adultes en salle d'opération, et aucune ne s'est spécifiquement intéressée aux chirurgies du sein. La sédation médicale (médicaments utilisés pour aider une personne à se détendre ou à s'endormir) pendant ce type de chirurgie a plusieurs objectifs : réduire l'anxiété, assurer le confort lors d'interventions plus longues et soulager les douleurs.

Notre étude vise à déterminer si l'utilisation d'un casque de réalité virtuelle peut diminuer les besoins en médicaments sédatifs pendant les chirurgies du sein réalisées chez des patientes de 18 ans et plus.

Pour cette recherche, nous voulons inclure 100 femmes, âgées de 18 ans ou plus, devant bénéficier d’une chirurgie du sein sous bloc paravertébral.

**DÉROULEMENT DU PROJET DE RECHERCHE**

Ce projet de recherche se déroulera au CIUSSS-CEMTL, hôpital Maisonneuve-Rosemont.

**1. Durée et nombre de visites**

Votre participation à ce projet de recherche commencera le matin de votre chirurgie (vous serez amenée à répondre à des questionnaires avec une personne de l’équipe de recherche) et se terminera le jour même après votre chirurgie.

L’équipe de recherche accédera à votre dossier médical pour recueillir les informations pertinentes pour la réalisation de cette étude.

**2. Dispositif à l’étude**

En participant à ce projet de recherche, vous serez assigné à l’un des groupes suivants :

**Groupe 1** : Soins standards et utilisation de la réalité virtuelle

**Groupe 2** : Soins standards

Ce projet de recherche est randomisé, ce qui signifie que vous serez assigné à l’un ou l’autre des deux groupes. Votre assignation à l’un ou à l’autre de ces groupes de traitement relève du hasard (comme tirer à pile ou face), vous ne pourrez donc pas choisir votre groupe. Ainsi, 1 personne sur 2 (50 %) utilisera la réalité virtuelle et 1 personne sur 2 (50 %) ne l’utilisera pas.

Toutes les patientes auront accès à une manette leur permettant de contrôler elles-mêmes la quantité de médicaments de sédation qu'elles souhaitent recevoir pour assurer leur confort, ce qu’on appelle la sédation contrôlée par le patient, sous la surveillance d'un anesthésiologiste. Cette stratégie de sédation serait utilisée, que vous acceptiez de participer à la recherche ou non. De même, on installera un capteur digital sur le doigt, appelé capteur de l’indice du niveau de nociception (NOL index), qui sert à détecter les niveaux de douleur et d’inconfort.

Par ailleurs la chirurgie se déroulera de façon standard. De plus, les soins qui vous seront donnés pendant et après la chirurgie ne seront pas modifiés par la participation à ce projet de recherche.

**3.Tests et procédures**

Durant votre participation à ce projet de recherche, le chercheur responsable de ce projet de recherche ou un membre de son personnel de recherche effectueront les interventions et analyses suivantes :

| **DESCRIPTION DES PROCÉDURES D’ÉTUDE** | |
| --- | --- |
| **Procédures** | **Description** |
| Questionnaires | On vous demandera de répondre à des questionnaires avec un des membres de l’équipe de recherche.    Les questions posées avant l’opération porteront sur votre niveau d’anxiété relativement à l’anesthésie, vos caractéristiques (ex : votre niveau d’éducation),votre utilisation précédente de la réalité virtuelle ainsi que votre enthousiasme à l’idée d’utiliser un casque de réalité virtuelle pendant la chirurgie.    Les questions posées suite à l’opération, si vous êtes dans le groupe 1 (réalité virtuelle), porteront sur la facilité d’utilisation, l’agréabilité ainsi que votre satisfaction générale par rapport à l’utilisation du casque de réalité virtuelle. |
| Utilisation du casque de réalité virtuelle (groupe 1 uniquement) | Juste avant votre chirurgie, on vous montrera trois vidéos présentant les trois choix de scénarios de réalité virtuelle et on vous demandera d’en choisir un.    Une fois l’anesthésie régionale réalisée, vous serez installée sur le dos et le masque de réalité virtuelle sera placé sur votre visage, puis le scénario que vous aurez choisi sera démarré. Si vous souhaitez changer de scénario pendant la chirurgie, vous pourrez le mentionner à un membre de l’équipe de recherche. |

**AVANTAGES ASSOCIÉS AU PROJET DE RECHERCHE**

Il se peut que vous retiriez un bénéfice personnel de votre participation à ce projet de recherche, mais nous ne pouvons vous l’assurer. Par ailleurs, nous espérons que les résultats obtenus contribueront à l’avancement des connaissances scientifiques dans ce domaine et au développement de meilleurs traitements pour les patients.

**RISQUES ET INCONVÉNIENTS ASSOCIÉS AU PROJET DE RECHERCHE**

Si vous constatez un effet secondaire, quel qu’il soit, au cours de ce projet, vous devez immédiatement avertir le chercheur responsable de ce projet de recherche, que vous croyiez ou non que cet effet soit en lien avec le casque de réalité virtuelle.

Le chercheur responsable de ce projet de recherche et les membres de son personnel de recherche répondront aux questions que vous pourriez avoir à ce sujet.

Voici les effets secondaires possibles de la réalité virtuelle avec leur risque de gravité. Ces effets secondaires, lorsqu’ils surviennent, sont de courte durée. Ils disparaissent généralement en 30 minutes.

| **EFFETS SECONDAIRES DE LA RÉALITÉ VIRTUELLE** | |
| --- | --- |
| Courants et bénins(plus de 10%) | Rares (moins de 1/1000 ou 0,1%) |
| - Inconfort - Fatigue oculaire - Nausée - Étourdissements - Désorientation - Vertiges - Maux de tête - Mal des transports | - Crise d’épilepsie |

**AUTRES TRAITEMENTS POSSIBLES**

Vous n’êtes pas obligée de participer à ce projet de recherche pour recevoir des soins médicaux pour votre condition. Nous vous invitons à parler au chercheur responsable de ce projet de recherche des diverses options disponibles.

**PARTICIPATION VOLONTAIRE ET DROIT DE RETRAIT**

Votre participation à ce projet de recherche est volontaire. Vous êtes donc libre de refuser d’y participer. Vous pouvez également vous retirer de ce projet de recherche à n’importe quel moment, sans avoir à donner de raisons, en informant le médecin responsable du projet de recherche ou un membre de l’équipe de recherche.

Votre décision de ne pas participer à ce projet de recherche ou de vous en retirer n’aura aucune conséquence sur la qualité des soins et des services auxquels vous avez droit ou sur votre relation avec les équipes qui les dispensent.

Le médecin responsable de ce projet de recherche ou le Comité d’éthique de la recherche du CIUSSS-EMTL peuvent mettre fin à votre participation, sans votre consentement. Cela peut se produire si de nouvelles découvertes ou informations indiquent que votre participation au projet de recherche n’est plus dans votre intérêt, si vous ne respectez pas les consignes du projet de recherche ou encore s’il existe des raisons administratives d’abandonner le projet.

Si vous vous retirez du projet de recherche ou si vous êtes retirée du projet, aucune autre donnée ne sera recueillie. À moins d’avis contraire de votre part, l’information déjà recueillie dans le cadre de ce projet de recherche sera néanmoins conservée, analysée ou utilisée pour assurer l’intégrité du projet de recherche, comme le précise ce document.

Toute nouvelle connaissance acquise durant le déroulement du projet de recherche qui pourrait avoir un effet sur votre décision de continuer à y participer vous sera communiquée rapidement.

**UTILISATION SECONDAIRE DES DONNÉES**

Si vous y consentez, vos données pourront être utilisées dans le cadre d’autres projets de recherche dans le domaine de l’anesthésie sous la responsabilité du chercheur responsable. L’utilisation de vos données dans le cadre d’autres projets de recherche se fera après que ces projets auront eu été approuvés par un Comité d’éthique de la Recherche (CÉR) et sera soumise aux mêmes conditions de conservation, règles et normes que pour le présent projet.

**CONFIDENTIALITÉ**

Durant votre participation à ce projet de recherche, le médecin responsable du projet ainsi que l’équipe de recherche recueilleront, dans un dossier de recherche, les renseignements vous concernant et nécessaires pour répondre aux objectifs scientifiques du projet de recherche.

Ces renseignements peuvent comprendre les informations contenues dans votre dossier médical, y compris votre identité, dont votre nom, votre sexe, votre date de naissance, votre état de santé passé et présent, vos habitudes de vie ainsi que les résultats des tous les tests, examens et procédures réalisés en vue de votre opération.

Toutes les données recueillies demeureront confidentielles dans les limites prévues par la loi. Vous ne serez identifiée que par un numéro de code. La clé du code reliant votre nom à votre dossier de recherche sera conservée par le médecin responsable de ce projet de recherche.

Pour assurer votre sécurité, un document témoignant de votre participation (i.e. ce formulaire d’information et de consentement) est versé dans votre dossier médical. Par conséquent, toute personne ou compagnie à qui vous donnerez accès à votre dossier médical aura accès à ces informations.

Ces données de recherche seront conservées pendant au moins 7 ans après la fin de l’étude par le médecin responsable de ce projet de recherche

Les données de recherche pourront être publiées ou faire l’objet de discussions scientifiques, mais ne permettront pas de vous identifier.

À des fins de surveillance, de contrôle, de protection, de sécurité, votre dossier de recherche ainsi que vos dossiers médicaux pourront être consultés par une personne mandatée par des représentants de l’établissement ou du Comité d’éthique de la recherche du CIUSSS de l’Est-de-l’Île-de-Montréal. Ces personnes et ces organismes adhèrent à une politique de confidentialité.

Vous avez le droit de consulter votre dossier de recherche pour vérifier les renseignements recueillis et les faire rectifier au besoin.

**COMPENSATION**

Vous ne recevrez pas de compensation financière pour votre participation à ce projet de recherche.

**EN CAS DE PRÉJUDICE**

Si vous deviez subir quelque préjudice que ce soit par suite de toute procédure reliée à ce projet de recherche, vous recevrez tous les soins et services requis par votre état de santé.

En acceptant de participer à ce projet de recherche, vous ne renoncez à aucun de vos droits et vous ne libérez pas le chercheur responsable de ce projet de recherche et l'établissement de leur responsabilité civile et professionnelle.

**INSCRIPTION DU PROJET DE RECHERCHE**

Vous trouverez une description de cet essai clinique sur le site <https://clinicaltrials.org>, comme l’exige la loi des États-Unis. Ce site Web ne contiendra pas de renseignements permettant de vous identifier. Tout au plus, le site Web comprendra un résumé des résultats. Vous pouvez effectuer des recherches sur ce site Web en tout temps.

**IDENTIFICATION DES PERSONNES-RESSOURCES**

Si vous avez des questions ou éprouvez des problèmes en lien avec le projet de recherche, ou si vous souhaitez vous en retirer, vous pouvez communiquer avec le chercheur responsable de ce projet de recherche ou avec une personne de l’équipe de recherche au numéro suivant : 514-252-3400 poste 3193.

Pour toute question concernant vos droits en tant que participant à ce projet de recherche ou si vous avez des plaintes ou des commentaires à formuler, vous pouvez communiquer avec le Commissaire aux plaintes et à la qualité des services du CIUSSS de l’Est-de-l’Île-de-Montréal au 514-252-3400, poste 3510.

**SURVEILLANCE DES ASPECTS ÉTHIQUES DU PROJET DE RECHERCHE**

Le comité d’éthique de la recherche du CIUSSS de l’Est-de-l’Île-de-Montréal a approuvé le projet et en assurera le suivi. Pour toute information, vous pouvez communiquer avec le secrétariat du Comité au 514-252-3400, poste 5708.

| **Titre du projet de recherche :** | Utilisation de la réalité virtuelle comme stratégie d'anxiolyse et d’épargne pharmacologique peropératoire chez les patientes subissant des chirurgies mammaires : l'essai clinique randomisé V-RAPS |
| --- | --- |

**SIGNATURES**

***Signature du participant***

J’ai pris connaissance du formulaire d’information et de consentement. On m’a expliqué le projet de recherche et le présent formulaire d’information et de consentement. On a répondu à mes questions et on m’a laissé le temps voulu pour prendre une décision. Après réflexion, je consens à participer à ce projet de recherche aux conditions qui y sont énoncées.

J’autorise l’équipe de recherche à avoir accès à mon dossier médical.

Utilisation secondaire des données (optionnel): J’accepte que mes données soient utilisées dans d’autres études dans le domaine de l’anesthésie sous la responsabilité du chercheur principal :

Oui □ Non □

Nom et signature du participant Signature Date

***Signature de la personne qui obtient le consentement***

J’ai expliqué au participant le projet de recherche et le présent formulaire d’information et de consentement et j’ai répondu aux questions qu’il m’a posées.

Nom et signature de la personne qui obtient le consentement Signature Date

**Appendix B** World Health Organization trial registration dataset

| **Data category** | **Information** |
| --- | --- |
| Primary Registry and Trial Identifying Number | ClinicalTrials.gov  NCT06522711 |
| Date of Registration in Primary registry | July 25^th^, 2024 |
| Secondary Identifying Numbers | Unique Protocol ID: 2025-3802 |
| Source(s) of Monetary or Material Support | CR-HMR and the Department of Anesthesiology and Pain Medicine and Paperplane Therapeutics |
| Primary Sponsor | Centre intégré universitaire de santé et des services sociaux (CIUSSS) de l’est de l’île-de-Montréal (CEMTL) |
| Secondary Sponsor(s) | None |
| Contact for Public Queries | Pascal Laferrière-Langlois  ([pascal.laferriere-langlois@umontreal.ca](mailto:pascal.laferriere-langlois@umontreal.ca)) |
| Contact for Scientific Queries | Pascal Laferrière-Langlois  ([pascal.laferriere-langlois@umontreal.ca](mailto:pascal.laferriere-langlois@umontreal.ca)) |
| Public Title | The Virtual Reality for Anxiolysis and Pharmacological Sparing (V-RAPS) randomized controlled trial |
| Scientific Title | Virtual reality as a strategy for intra-operatory anxiolysis and pharmacological sparing in patients undergoing breast surgeries: the V-RAPS randomized controlled trial |
| Countries of Recruitment | Canada |
| Health Condition(s) or Problem(s) Studied | Intraoperative anxiety and need for sedation during elective breast surgeries in adult patients |
| Intervention(s) | Virtual reality immersion |
| Key Inclusion and Exclusion Criteria | Inclusion criteria: Full consent, aged 18 years or older, undergoing elective awake breast surgery under paravertebral block  Exclusion criteria: Hearing or visual impairment. history of epilepsy, seizure or severe dizziness, severe mental impairment, recent eye or facial surgery or wounds, inability to use the VR hand controller. |
| Study Type | Interventional, randomized, open-label, parallel, superiority study. |
| Date of First Enrollment | October 31^st^, 2024 (Anticipated) |
| Sample Size | 100 participants (Anticipated) |
| Recruitment Status | Not yet recruiting |
| Primary Outcome(s) | Average self-administration of propofol in mcg/kg/min |
| Key Secondary Outcomes | Level of anxiety before the surgery evaluated by the Amsterdam Preoperative Anxiety and Information Scale.  Incidence of adverse events.  Administration of fentanyl in mcg/kg.  Administration of ketamine in mg/kg.  Total duration in minutes spent by the patient on the VR scenario chosen, and the order in which VR scenarios were presented.  Post-anesthesia care unit length of stay  Ease of use of the technology and enjoyment of the first scenario chosen assessed post-operatively on a 10-point Likert scale in the intervention group only.  Overall satisfaction with the experience assessed post-operatively on a 10-point Likert scale in both groups. |
| Ethics Review | Approved by the regional ethics committee (Comité d’éthique en recherche - CIUSSS de l’Est de l'Île de Montréal) on September 9^th^, 2024 |
| Completion date | October 30^th^, 2026 (Anticipated) |
| Summary Results | None |
| IPD sharing statement | Individual clinical trial participant-level data will be made available upon request to the corresponding author, while maintaining strict patient confidentiality. |
