# Supplementary material for: Virtual reality as a strategy for intra-operatory anxiolysis and pharmacological sparing in patients undergoing breast surgeries: The V-RAPS randomized controlled trial protocol
Source: PLoS One. 2025 Jul 7;20(7):e0327555. doi: 10.1371/journal.pone.0327555 (PMC12233254; doi:10.1371/journal.pone.0327555)
Supplement: S1 File — (DOCX) [file pone.0327555.s002.docx]

**Virtual reality as a strategy for intra-operatory anxiolysis and pharmacological sparing in patients undergoing breast surgeries : the V-RAPS randomized controlled trial**

**Short title : Virtual Reality for Anxiolysis and Pharmacological Sparing (V-RAPS)**

Joe Zako MD^1^, Nicolas Daccache MD^1^, Julien Burey MD^1,2,3^, Ariane Clairoux, MD ^1^, Louis Morisson MD, MSc^1,2^, Pascal Laferrière-Langlois MD, MSc ^1,2^

*^1^ Department of Anesthesiology and Pain Medicine, Maisonneuve-Rosemont Hospital, Centre intégré universitaire de santé et service sociaux de l’Est de L'Île de Montréal, Montréal, Québec, Canada
^2^ Maisonneuve-Rosemont Hospital Research Center, Montréal, Québec, Canada
^3^ Department of Anesthesiology and Critical Care Medicine, Tenon Hospital, APHP Sorbonne Université, Paris, France*

Corresponding author : **Pascal Laferrière-Langlois**, Department of Anesthesiology and Pain Medicine, Maisonneuve-Rosemont Hospital, Centre intégré universitaire de santé et service sociaux de l’Est de L'Île de Montréal. 5415 boulevard de l'Assomption, Montréal, Québec H1T 2M4, Canada

Phone number : +1-819-432-5847
Email : [pascal.laferriere-langlois@umontreal.ca](mailto:pascal.laferriere-langlois@umontreal.ca)

ORCiD : 0000-0003-3347-1581

Authors:

Joe Zako : [joe.zako@umontreal.ca](mailto:joe.zako@umontreal.ca)

Julien Burey : [julien.burey@umontreal.ca](mailto:julien.burey@umontreal.ca) (0000-0003-2792-9883)

Nicolas Daccache : [nicolas.daccache@umontreal.ca](mailto:nicolas.daccache@umontreal.ca)

Airane Clairoux : [ariane.clairoux@umontreal.ca](mailto:ariane.clairoux@umontreal.ca)

Louis Morisson : [louis.morisson@umontreal.ca](mailto:louis.morisson@umontreal.ca) (0000-0003-4431-8785)

Fundings: This study will be supported by funds from Dr Pascal Laferrière-Langlois at the CR-HMR and the Department of Anesthesiology and Pain Medicine.

Declaration of interests:

Dr PLL declares ownership interest in private companies unrelated to this work (Divocco Medical and Divocco AI).

Other authors declare no competing interests.

Word count:

We used the SPIRIT checklist when writing our report (Chan et al., 2013)

Protocol version: 2, September 4th, 2024

# Article summary:

**Abstract:**

**Introduction:** Virtual reality (VR) has carved out a growing place for itself in our leisure activities, video games and even in medicine. Recent studies show that VR can reduce anxiety and pain in patients undergoing uncomfortable care (e.g. wound care, dental care, venous access). While the population most frequently investigated is pediatric, the benefits can be extended to other populations. The operating room is uncommon due to the type of procedure and the older patient profile. Yet, with the democratization of regional and neuraxial anesthesia, VR could improve the patient experience in the operating room.

**Objective:** The main objective of this study is to explore the impact of VR on intravenous (I.V.) sedation requirements in adult patients undergoing breast surgery under paravertebral (PV) block. The hypothesis is that VR will serve as an effective distraction in the operative context, reducing the need for pharmacology. In parallel, multiple secondary objectives will also be explored, such as the evaluation of the tolerance to the VR headset, the impact of the chosen type of scenario on the primary outcome, the incidence of adverse effects, the variation of the Nociception Level Index (NOL index) in awake patients as well as overall patient satisfaction.

**Material and methods:** This single center, open-label, randomized controlled trial will be conducted in a Canadian academic hospital. 100 patients above the age of 18 years undergoing breast surgery (mastectomy, reduction, augmentation, reconstruction, etc.) under regional anesthesia, specifically paravertebral block, will be included in this study. Patients will be randomized either to the intervention group or the control group. The intervention group will have a VR helmet and the control group a standard treatment. Both will have access to patient-controlled sedation (PCS), self-administering their own sedation under clinical observation. Patients in the VR group will be able to choose, before the procedure begins, between 3 different VR environments and will also be allowed to switch between these environments during the surgery. The primary outcome of this study will be the time-weighted dose of propofol (sedative) self-administered during the procedure. Secondary outcomes will include patient satisfaction, premature removal of VR helmet, presence of nausea, desaturation, hypotension and post-anesthesia monitoring time.

**Ethics considerations:** This trial will be submitted to the regional ethics committee; approval is pending.

**Study registration:** Clinicaltrials.gov (pending)

**Keywords:** virtual reality, breast surgery, anxiety, pain, NOL index, patient-controlled sedation, propofol

**Strengths and Limitations of this study:**

- Multimodal and innovative pain management approach
- Potential for pharmacological sparing
- Quantifiable outcomes
- Single center design
- Short-term outcomes

# Introduction

## **Background and rationale**

For the past decade Virtual Reality (VR) has been expanding across various medical application fields. It is based on the computer-generated three-dimensional environment which the end-user can interact with. Interfaces used to access this virtual world determine the level of immersion in such experiences, ranging from a smartphone screen that projects the world through its camera, to virtual reality headsets, and up to fully immersive solutions that provide haptic feedback [(Boutin et al., 2023)](https://www.zotero.org/google-docs/?t3c1Ic), enabling the simulation of touch in addition to sight and hearing. The more senses VR involves, the more immersive it becomes.

There are several types of VR used in healthcare, depending, among other things, on the degree of interaction with the real world. Augmented reality enables professionals or patients to add dynamic, interactive virtual elements to their environment. Some surgical teams integrate it into intraoperative procedures, where a 3 dimensional reconstruction of the patient's anatomy, based on medical imaging data, is superimposed on the patient, and guides surgeons throughout the surgery [(Ryu et al., 2022)](https://www.zotero.org/google-docs/?5CCNjD).

Immersive Virtual Reality (IVR) requires the use of specific equipment such as a headset, combined with interface elements that can be a console controller, or specific hardware recognized by the headset and allowing the user to manipulate elements of the virtual environment. This solution is already used in the initial phase of medical education for residents [(Kuhn et al., 2024)](https://www.zotero.org/google-docs/?Zuhb4o). Indeed, the development of this type of technology alongside other simulation tools has ensured that students do not perform procedures for the first time on a real patient. This guarantees the safety of the patient, builds the confidence of the person performing the act, and enables a better mentoring process. This is particularly true for surgical procedures, where virtual reality simulation enables risky maneuvers to be rehearsed [(Yi et al., 2024)](https://www.zotero.org/google-docs/?OrIW00). Robotic surgery offers immersive experiences very close to reality, and training programs on these machines are multiplying. Similarly, IVR integrates perfectly with other promising technologies such as digital twins. Surgical teams report repeating complex surgeries in advance to optimize high-risk operating times or for exceptional cases [(Chumnanvej et al., 2024)](https://www.zotero.org/google-docs/?JzMNar).

Another application for VR is to reduce anxiety and improve patient comfort in the perioperative and periprocedural periods. Several teams have validated the use of VR headsets in pediatric populations [(Eijlers et al., 2019; Wang et al., 2022)](https://www.zotero.org/google-docs/?koBih3).

Regarding surgery and anesthesia, VR was used during different procedures such as orthopedic surgeries of the upper extremities under regional anesthesia blocks, hip and knee arthroplasties, dental care and induction of anesthesia, especially in pediatrics [(Boyce et al., 2023; Hitching et al., 2023)](https://www.zotero.org/google-docs/?VMQ42e). Using VR during surgery has been shown to reduce pain, anxiety, quantity of sedation used and time in the PACU. In fact, some research shows that certain types of VR media might be superior to others. For example, a recently published trial has shown that VR video games seem to increase the pain threshold and reduce anxiety more than a simple natural landscape VR presentation during dental surgery (Yamashita et al., 2023). It can be hypothesized from these results that active, rather than passive, immersion in a VR environment creates greater distractibility, leading to better analgesia and anxiolysis. Moreover, a recently published study attempted to evaluate the effects of immersive virtual reality therapy on adult patients undergoing orthopedic surgery with access to patient-controlled sedation (PCS) (Huang et al., 2020). Although this study showed no difference in propofol usage between the intervention and control group, it effectively opened the door for further similar research in order to increase the total sample size and lead to an eventual meta-analysis.

Concerning PCS, it is a therapeutic modality that began gaining popularity in the late 20th century (Rudkin et al., 1991). However, it has only recently been validated as a safe equivalent, and maybe even superior, alternative method when compared to clinician-controlled sedation, with potentially lower rates of hypotension and oxygen desaturation as well as reduced recovery times and lessened risk of rescue interventions (Lu et al., 2015; Kreienbühl et al., 2018). As these patients self-administer their own pharmaceuticals during awake surgeries under constant anesthesiological supervision, it makes for an excellent and secure method in assessing the difference in sedation requirements between two patient groups while reducing the risk of clinician performance bias in the case of a non-blinded trial.

Finally, about the Nociception Level Index (NOL Index), it is a new technology developed by the company *Medasense* with the goal of creating an objective way of measuring pain, or rather nociception in patients under general anesthesia, making it possible to more accurately administer analgesia and sedation without exceeding the necessary amount. It does so with the use of a digital sensor that detects skin temperature, small movements, galvanic skin responses all while performing a constant photoplethysmography. This technology has only been validated in unconscious patients under general anesthesia and has shown a significant reduction in postoperative pain scores in patients having benefited from its use (Fuica et al., 2023). However, it has also recently been tested on awake and alert postoperative cardiac surgery patients in the intensive care unit and has been shown to be able to discriminate between nociceptive and non-nociceptive stimuli; it also correlated well with the self-reported pain and feeling of discomfort occurring during chest tube removal (Gélinas et al., 2021).

**Objectives**

Primary Objective

The main objective of this study is to determine if intraoperative virtual reality immersion can reduce propofol requirements for female patients undergoing breast surgery under paravertebral block.

Secondary Objectives

Secondary objectives are the following:

- Evaluate the level of anxiety before the surgery.
- Evaluate the differences in sedation requirements depending on the type of VR scenario.
- Evaluate quantities and distribution over time of remifentanil administered.
- Evaluate quantities of ketamine administered.
- Evaluate the incidence of adverse effects such as cybersickness, nausea, bradycardia, desaturation and hypotension.
- Evaluate the time the patient spent wearing the headset
- Evaluate the requirement of post-operative care and the post-anesthesia monitoring time.
- Appreciate the participants’s initial enthusiasm at the idea of using a VR headset during surgery, ease of use of the technology, enjoyment of the first scenario chosen and overall satisfaction with the experience.
- Explore the use of the NOL index in awake patients undergoing surgery.

The main hypothesis of our study is that female patients undergoing breast surgery under regional anesthesia with an immersive VR experience will require on average **30% less propofol** than the control group. We also hypothesize that interactive scenarios will further reduce the requirement for sedation, when compared to non-interactive scenarios.

## **Study design**

This is a minimal-risk, open-label randomized controlled trial, conducted at a single center.

# Methods: Participants, interventions, and outcomes

# **Study setting**

This research project will take place at Maisonneuve-Rosemont Hospital, part of the Centre Intégré Universitaire de Santé et des Services Sociaux (CIUSSS) de l’est de l’île-de-Montréal (CEMTL), located in Montreal, Quebec, Canada. Participant recruitment will take place from August 2024 to August 2025 and experimentation and data collection from August 2024 to October 2025.

## **Eligibility criteria**

We will screen and aim to recruit consecutive adult female patients undergoing elective, awake, breast surgery requiring a paravertebral block.

Inclusion criteria

- Fully consented female patients above the age of 18 years;
- Undergoing elective, awake, breast surgery performed under paravertebral block.

Exclusion criteria

- Hearing or visual impairment;
- History of epilepsy, seizure or severe dizziness;
- Severe mental impairment;
- Recent eye or facial surgery or wounds;
- Inability to use the VR hand controller.

## **Randomization**

After the aforementioned patients meet all the inclusion criteria and meet no exclusion criteria, patients will then be randomized by an unbiased algorithm in a 1 for 1 ratio (one control patient for each patient in the VR group). However, the chosen scenario in the VR group will be a result of each patient’s personal preference; the patients will also be able to switch between scenarios at will.

## **Interventions**

Prior to their arrival in the operating room, all participants who met inclusion criteria, were recruited and have signed the consent form, will be randomized and provided explanation on the use of intraoperative patient-controlled sedation. Specifically in the intervention group, a brief overview of all three VR scenarios will be shown in a three separate videos prior to letting the participant select the preferred scenario. The participant’s choice of scenario will then be documented.

Upon entry in the operating room, all participants will undergo routine monitoring according to the Canadian Standards Association (CSA) guidelines, which will encompass non-invasive blood pressure, pulse-oximetry, and EKG. The monitoring will be conducted using the Dräger Infinity C700 (Dräger Medical, Lübeck, Germany) monitor. Throughout the entire anesthesia duration, the NOL index finger probe (PMD-200 device, Medasense Biometrics Ltd, Ramat Gan, Israel) will be applied. All intraoperative data and events will be recorded on the research computer. Paravertebral blocks will be performed by the attending anesthesiologist as standard care.

Once paravertebral blocks are completed and the participants are in supine position and the surgeon is ready the disinfection, the VR headset will be applied on the face of the participants in the intervention group and the chosen scenario will be started. If at any point the patient expresses a desire to stop using VR during the surgery, we will first offer them an alternate scenario. If they reiterate their willingness to stop, with or without having experienced the new scenario, the headset will be removed for the remainder of the surgery.

## **Outcomes**

Primary outcome:

The primary endpoint is the time-adjusted and weight-adjusted average self-administration of propofol in mcg/kg/min in female patients undergoing breast surgery under paravertebral block.

Secondary outcomes:

- Level of anxiety before the surgery evaluated by the Amsterdam Preoperative Anxiety and Information Scale.
- Time-adjusted and weight-adjusted average administration of remifentanil in mcg/kg/min.
- Weight-adjusted administration of ketamine in mg/kg.
- The incidence of adverse events such as. bradycardia, desaturation and hypotension. Subjective adverse events, such as cybersickness or nausea, will also be assessed as spontaneously reported by the participants.
- Percentage of patients that switched scenarios.
- The total time, in minutes, as well as the relative time compared to the duration of the surgery, expressed as a percentage, during which the VR headset was worn.
- Total duration in minutes spent by the patient on the VR scenario chosen, and the order in which VR scenarios were presented.
- Percentage of patients that removed the headset before the end of the surgery.
- The total time in minutes spent in the PACU.
- Initial enthusiasm at the idea of using a VR headset during surgery will be assessed on a 10-point Likert scale for all participants, prior to randomization (as part of a self-reported pre-operative questionnaire) Ease of use of the technology, enjoyment of the first scenario chosen and overall satisfaction with the experience will be assessed post-operatively on a 10-point Likert scale in the intervention group only (as part of a self-reported post-operative questionnaire).
- NOL index readings over time per patient, assessed continuously.

Patients' socio-demographic information, including age, weight, height, sex, their main medical history and medications will be collected from patients’ medical charts. Their education level and familiarity with and/or regular use of VR will also be collected (as part of a self-reported pre-operative questionnaire).

## **Participant timeline and recruitment**

The research team will assess the elective surgical list of Maisonneuve-Rosemont Hospital’s operating room to identify eligible patients at least one week before their scheduled surgery.

Potential patients will be contacted by the research team via phone to provide an explanation of the project. The communication form used during these calls will be approved by the CIUSSS EMTL Research Ethics Committee.

A thorough review of the patients’ medical charts will be conducted to confirm that they meet the inclusion criteria and have no exclusion criteria. Subsequently, if they express interest in participating in this study, the research team will meet the candidates before the surgery to address any questions they may have. They will then be asked to sign the consent form and complete a pre-operative questionnaire prior to randomization.

Once in the operating room, the anesthesia and surgery will be performed with or without the VR headset, depending on randomization. The patient’s involvement will be completed at the discharge from the PACU.

## **Sample size**

Available data in the literature is scarce concerning sedation requirements in individuals with intraoperative VR headsets during surgeries under regional anesthesia. Some trials reported a significant reduction from 708 (+/- 293 mg) to 48 mg (+/- 27 mg), while other trials reported no benefits. In this trial, we expect a reduction of propofol usage by 30% in the VR group when compared to the control group. At a confidence level of 95%, a power of 80%, and a normalized standard deviation of 0.5, a total of 90 patients are required to have the precision needed on our tolerance point estimate. To account for a potential dropout ratio of 10.0%, we inflated the number to **100** patients.

# Methods: Assignment of interventions

## **Allocation**

Electronic randomization will be performed by a statistician using a computer-generated randomized sequence with a variable block size unknown to the investigators, and then implemented in a RedCAP application. It will be sealed in an opaque envelope and handed to the dedicated research staff not involved in patient care. The envelope will be opened at the patient's entry into the operating ward, after confirming that the surgery will be performed. Once the group is assigned to the patient, the research staff will prepare the VR gear and present short videos pertaining to the three possible VR scenarios available, after which the intervention group patients will get to pick their initial scenario. If a patient wants to change scenarios during the surgery, the dedicated research staff will be responsible for operating this change. If the patient wants to remove the headset, the research staff will first offer a new scenario to the patient. If the patient refuses the new scenario or wants to remove the headset during the new scenario, the VR gear will be removed for the rest of the surgery.

Due to the nature of the intervention, the patient, the anesthesiologist and the surgeon cannot be blinded to the randomization. However, the PACU staff as well as the research team acting as outcome assessors will be blinded to the randomization.

In the event of adverse events attributable to VR gear (for example, severe cybersickness), discontinuation of the VR immersion will rest at the discretion of the anesthesiologist in charge of the patient as well as the patient. These events will be collated and analyzed subsequently.

# Methods: Data collection, management, and analysis

## **Data collection methods**

Electronic data from medical monitoring device PMD-200, will be collected. The time of intraoperative events will be manually recorded. A designated research computer will gather all the data electronically and will use the integrated events tag system of the Medasense PMD-200, or the BetterCare software (provided by Dräger, Lübeck, Germany) connected to the Dräger anesthesia workstation.

All anesthesia and surgery-related events, the preoperative questionnaires as well as postoperative assessments will be recorded separately on a Case Report Form (CRF). The CRF will be built using REDCap software (Vanderbilt University), which will be accessed via electronic tablets by the research team in the operating room. Data will be electronically extracted under a unique identification number assigned at enrollment, to a secured, offline research computer, ensuring patient anonymization. Patient identity will only be mentioned on the consent form, which will be kept in a locked closet in the Department of Anesthesiology at Maisonneuve-Rosemont Hospital for 7 years.

## **Data management**

Protected health information will not be re-used or disclosed to a third party except as required by law, for authorized oversight of the research, or as permitted by an authorization signed by the research subject.

Data will be stored on a password-protected laptop which has been assigned to Dr Pascal Laferrière-Langlois. He will maintain primary responsibility for this computer and the data it contains.

Paper copies (consent form) of patient information will be stored in a locked file cabinet in a locked office in the Department of Anesthesiology. Data will be assigned a unique study code (P1, P2, and so on up until PX) which will be linked to the subject’s identities. The link will be kept separately from the data so that no patient can be identified in the event of loss or theft.

Only the principal investigator and the co-investigators will have access to all the data during the study and study analysis. All records relating to this clinical trial will be retained for a period of 7 years.

Every person involved in this study will receive appropriate training and abide by confidentiality guidelines to protect the subject’s privacy. All applicable laws, rules and regulations will be strictly followed.

**Statistical methods**

Descriptive statistics will be presented by groups using mean and standard deviation, or median and interquartile range if the distribution is skewed or not normal, for continuous parameters (according to the skewness of the distribution of each parameter). Frequency (%) will be used for categorical parameters. According to the nature of the analyzed endpoints, confidence intervals (CIs) between proportions or between means/medians differences will be presented. Risk differences or mean differences with 95% confidence intervals will be reported for all outcomes. The alpha value will be set at 0.05 to establish statistical significance. The following table represents the statistical tests that will be used depending on the distribution and variances of the results obtained.


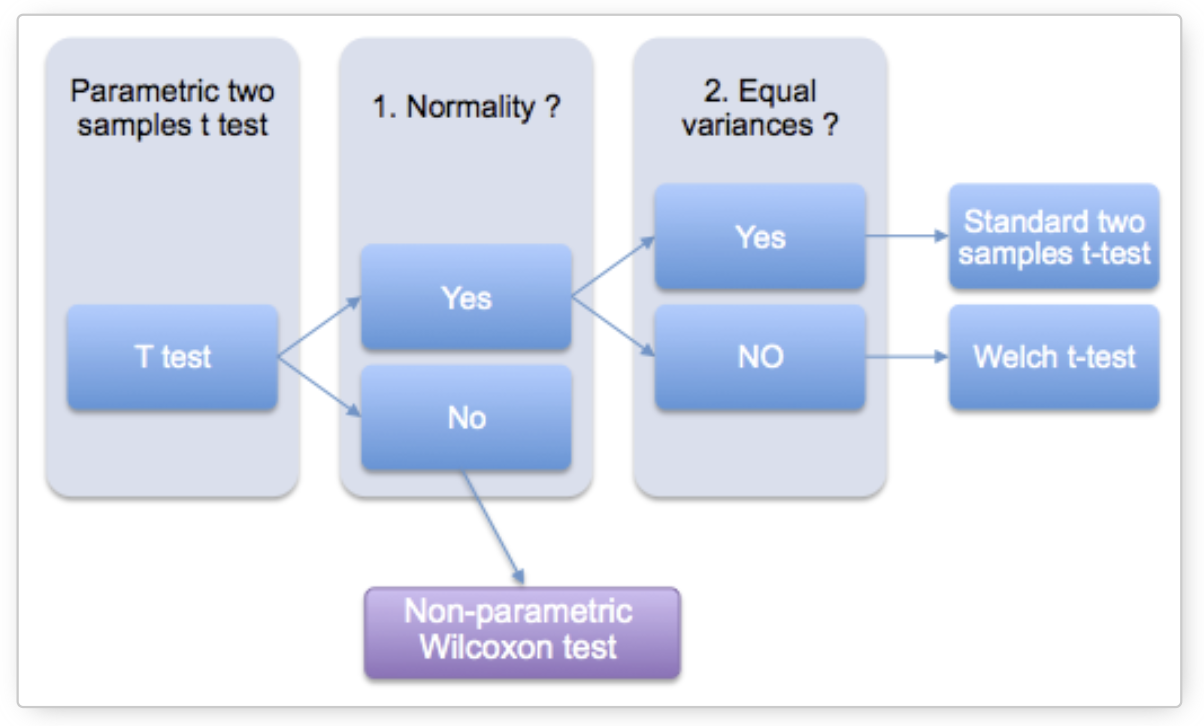


Univariate analyses will be performed to examine the relationship between the primary outcome and other potentially dependent variables. Each independent variable will be analyzed individually in relation to the primary outcome using logistic regression. If relevant following univariate analysis, multivariate analysis will be performed to examine the impact of potential independent variables.

We will perform subgroup analysis to assess a number of potential influences on our results. While the effect of age, for example, will be captured by univariate analysis, we will specifically conduct subgroup analysis of our primary and secondary outcomes by segmenting our trial population with the chosen VR scenario. This will enable the exploration of our secondary objectives.

All statistical analysis will be performed using SAS, SPSS software or python programming language via VS Code or Jupyter notebook software.

# Methods: Monitoring

## **Data monitoring**

The study has a low level of risk. The Principal Investigator (PI) or designated personnel will regularly review all data, such as study completeness, enrollment, protocol deviations, dropouts, and adverse events, on a weekly or bi-weekly basis.

## **Harms**

Ensuring subject safety is a top priority for the research team and hospital staff. In the unlikely chance of an unexpected adverse event, the principal investigator, Dr. Pascal Laferrière-Langlois, will be immediately notified. Prompt actions will be taken to provide appropriate care.

The devices used in this study include a VR headset wirelessly paired with a tablet in order to broadcast immersive scenarios. Its use does not impact intraoperative monitoring and the occurrence of an adverse event associated with its use is highly unlikely.

Subject safety will be always guaranteed by research and hospital staff during the completion of the study procedures. All adverse effects will be recorded and patients at risk of serious side effects will be screened and excluded from the present study.

## **Auditing**

There will be no formal plan for auditing or inspecting.

# Ethics and dissemination

## **Research ethics approval**

This protocol will been submitted to CER-CEMTL (Comité d’éthique en recherche - CIUSSS de l’Est de l'Île de Montréal) for approval.

Paperplane Therapeutics, which provides the VR headset and solution, is currently seeking Health Canada’s approval.

This trial will be registered on Clinicaltrials.gov and made publicly available (pending).

## **Protocol amendments**

Any major modification to the protocol will be published on the Clinicaltrials.gov.

## **Consent**

Potential patients will be contacted by the research team via phone to provide an explanation of the project. The communication form used during these calls will be approved by the CIUSSS EMTL Research Ethics Committee. The research team will meet the candidates before the surgery to address any questions they may have. If the patients still wish to participate, informed consent will be obtained and signed.

We will track the number of withdrawn subjects. One possible reason a subject may decide to withdraw from the trial is a desire to discontinue their participation. Therefore, we will remove a participant if they mention to the research personnel that they no longer wish to continue with the study. We will not store data or follow-up after participant withdrawal.

## **Declaration of interests**

Dr PLL declares ownership interest in private companies unrelated to this work (Divocco Medical and Divocco AI).

Other authors declare no competing interests.

## **Dissemination policy**

We plan to publish the results of this trial in a mid-impact factor journal in the field of anesthesia, or medical technology.

Patients or the public were not involved in the design, or conduct, or reporting, or dissemination plans of our research.

Full protocol as well as supplementary data from this study could be made available upon request to the PI.

To be eligible for authorship, contributors must have significantly participated in the creation, development, or enrollment of participants or statistical analysis of the study.

## **Acknowledgements**

Thanks to all the research team at LIAM (Laboratory of innovative anesthesia in Montreal) for its help in the research organization.

**Author contributions**

JZ & JB were involved in the study conception and design, literature research, and manuscript draft.

LM was involved in the study conception and design, statistical planning, and manuscript revision.

OV & ND was involved in the study conception and design, and manuscript revision.

PLL was involved in the study conception and design, literature research, and manuscript revision.

All authors read and approved the final manuscript. The corresponding author had full access to all the data in the study and had final responsibility for the decision to submit for publication.

# References

[Boutin, J., Kamoonpuri, J., Faieghi, R., Chung, J., de Ribaupierre, S., Eagleson, R., 2023. Smart haptic gloves for virtual reality surgery simulation: a pilot study on external ventricular drain training. Front. Robot. AI 10, 1273631. https://doi.org/10.3389/frobt.2023.1273631](https://www.zotero.org/google-docs/?rQxiqb)

[Boyce, L., Jordan, C., Egan, T., Sivaprakasam, R., 2023. Can virtual reality enhance the patient experience during awake invasive procedures? A systematic review of randomized controlled trials. Pain. https://doi.org/10.1097/j.pain.0000000000003086](https://www.zotero.org/google-docs/?rQxiqb)

[Chumnanvej, Sorayouth, Chumnanvej, Siriluk, Tripathi, S., 2024. Assessing the benefits of digital twins in neurosurgery: a systematic review. Neurosurg. Rev. 47, 52. https://doi.org/10.1007/s10143-023-02260-5](https://www.zotero.org/google-docs/?rQxiqb)

[Eijlers, R., Utens, E.M.W.J., Staals, L.M., de Nijs, P.F.A., Berghmans, J.M., Wijnen, R.M.H., Hillegers, M.H.J., Dierckx, B., Legerstee, J.S., 2019. Systematic Review and Meta-analysis of Virtual Reality in Pediatrics: Effects on Pain and Anxiety. Anesth. Analg. 129, 1344–1353.](https://www.zotero.org/google-docs/?rQxiqb) <https://doi.org/10.1213/ANE.0000000000004165>

Fuica, R., Krochek, C., Weissbrod, R., Greenman, D., Freundlich, A., & Gozal, Y., 2023. Reduced postoperative pain in patients receiving nociception monitor guided analgesia during elective major abdominal surgery: a randomized, controlled trial. Journal of clinical monitoring and computing, 37(2), 481–491. https://doi.org/10.1007/s10877-022-00906-1

Gélinas, C., Shahiri T, S., Richard-Lalonde, M., Laporta, D., Morin, J. F., Boitor, M., Ferland, C. E., Bourgault, P., & Richebé, P., 2021. Exploration of a Multi-Parameter Technology for Pain Assessment in Postoperative Patients After Cardiac Surgery in the Intensive Care Unit: The Nociception Level Index (NOL)TM. Journal of pain research, 14, 3723–3731. https://doi.org/10.2147/JPR.S332845

[Hitching, R., Hoffman, H.G., Garcia-Palacios, A., Adamson, M.M., Madrigal, E., Alhalabi, W., Alhudali, A., Sampaio, M., Peterson, B., Fontenot, M.R., Mason, K.P., 2023. The Emerging Role of Virtual Reality as an Adjunct to Procedural Sedation and Anesthesia: A Narrative Review. J. Clin. Med. 12, 843. https://doi.org/10.3390/jcm12030843](https://www.zotero.org/google-docs/?rQxiqb)

[Huang, M.Y., Scharf, S., Chan, P.Y., 2020. Effects of immersive virtual reality therapy on intravenous patient-controlled sedation during orthopaedic surgery under regional anesthesia: A randomized controlled trial. PloS One 15, e0229320.](https://www.zotero.org/google-docs/?rQxiqb) <https://doi.org/10.1371/journal.pone.0229320>

Kreienbühl, L., Elia, N., Pfeil-Beun, E., Walder, B., & Tramèr, M. R., 2018. Patient-Controlled Versus Clinician-Controlled Sedation With Propofol: Systematic Review and Meta-analysis With Trial Sequential Analyses. Anesthesia and analgesia, 127(4), 873–880. https://doi.org/10.1213/ANE.0000000000003361

[Kuhn, A.W., Yu, J.K., Gerull, K.M., Silverman, R.M., Aleem, A.W., 2024. Virtual Reality and Surgical Simulation Training for Orthopaedic Surgery Residents: A Qualitative Assessment of Trainee Perspectives. JB JS Open Access 9, e23.00142.](https://www.zotero.org/google-docs/?rQxiqb) <https://doi.org/10.2106/JBJS.OA.23.00142>

Lu, Y., Hao, L. X., Chen, L., Jin, Z., & Gong, B., 2015. Systematic review and meta-analysis of patient-controlled sedation versus intravenous sedation for colonoscopy. International journal of clinical and experimental medicine, 8(11), 19793–19803.

Rudkin, G. E., Osborne, G. A., & Curtis, N. J., 1991. Intra-operative patient-controlled sedation. Anaesthesia, 46(2), 90–92. https://doi.org/10.1111/j.1365-2044.1991.tb09345.x

[Ryu, S., Kitagawa, T., Goto, K., Okamoto, A., Marukuchi, R., Hara, K., Ito, R., Nakabayashi, Y., 2022. Intraoperative Holographic Guidance Using Virtual Reality and Mixed Reality Technology During Laparoscopic Colorectal Cancer Surgery. Anticancer Res. 42, 4849–4856. https://doi.org/10.21873/anticanres.15990](https://www.zotero.org/google-docs/?rQxiqb)

[Wang, Y., Guo, L., Xiong, X., 2022. Effects of Virtual Reality-Based Distraction of Pain, Fear, and Anxiety During Needle-Related Procedures in Children and Adolescents. Front. Psychol. 13, 842847.](https://www.zotero.org/google-docs/?rQxiqb) <https://doi.org/10.3389/fpsyg.2022.842847>

Yamashita, Y., Aijima, R., & Danjo, A., 2023. Clinical effects of different virtual reality presentation content on anxiety and pain: a randomized controlled trial. Scientific reports, 13(1), 20487. https://doi.org/10.1038/s41598-023-47764-8

[Yi, W.S., Rouhi, A.D., Duffy, C.C., Ghanem, Y.K., Williams, N.N., Dumon, K.R., 2024. A Systematic Review of Immersive Virtual Reality for Nontechnical Skills Training in Surgery. J. Surg. Educ. 81, 25–36. https://doi.org/10.1016/j.jsurg.2023.11.012](https://www.zotero.org/google-docs/?rQxiqb)
