# Supplementary material for: Virtual reality as a strategy for intra-operatory anxiolysis and pharmacological sparing in patients undergoing breast surgeries: The V-RAPS randomized controlled trial protocol
Source: PLoS One. 2025 Jul 7;20(7):e0327555. doi: 10.1371/journal.pone.0327555 (PMC12233254; doi:10.1371/journal.pone.0327555)
Supplement: Attachment — Submitted filename: VRAPS REVIEW COMMENTS.pdf [file pone.0327555.s005.pdf]

Comments to the Author

1. Does the manuscript provide a valid rationale for the proposed study, with clearly identified and justified research questions?

The research question outlined is expected to address a valid academic problem or topic and contribute to the base of knowledge in the field.

Reviewer #1: Yes

Reviewer #2: Yes

Reviewer #3: Yes

2. Is the protocol technically sound and planned in a manner that will lead to a meaningful outcome and allow testing the stated hypotheses?

The manuscript should describe the methods in sufficient detail to prevent undisclosed flexibility in the experimental procedure or analysis pipeline, including sufficient outcome-neutral conditions (e.g. necessary controls, absence of floor or ceiling effects) to test the proposed hypotheses and a statistical power analysis where applicable. As there may be aspects of the methodology and analysis which can only be refined once the work is undertaken, authors should outline potential assumptions and explicitly describe what aspects of the proposed analyses, if any, are exploratory.

Reviewer #1: Yes

Reviewer #2: Partly

Reviewer #3: Yes

3. Is the methodology feasible and described in sufficient detail to allow the work to be replicable?

Descriptions of methods and materials in the protocol should be reported in sufficient detail for another researcher to reproduce all experiments and analyses. The protocol should describe the appropriate controls, sample size calculations, and replication needed to ensure that the data are robust and reproducible.

Reviewer #1: Yes

Reviewer #2: No

Reviewer #3: Yes

4. Have the authors described where all data underlying the findings will be made available when the study is complete?

The PLOS Data policy requires authors to make all data underlying the findings described in their manuscript fully available without restriction, with rare exception, at the time of publication. The data should be provided as part of the manuscript or its supporting information, or deposited to a public repository. For example, in addition to summary statistics, the data points behind means, medians and variance measures should be available. If there are restrictions on publicly sharing data—e.g. participant privacy or use of data from a third party—those must be specified.

Reviewer #1: Yes

Reviewer #2: Yes

Reviewer #3: Yes

5. Is the manuscript presented in an intelligible fashion and written in standard English?

PLOS ONE does not copyedit accepted manuscripts, so the language in submitted articles must be clear, correct, and unambiguous. Any typographical or grammatical errors should be corrected at revision, so please note any specific errors here.

Reviewer #1: Yes

Reviewer #2: Yes

Reviewer #3: Yes

6. Review Comments to the Author

Please use the space provided to explain your answers to the questions above and, if applicable, provide comments about issues authors must address before this protocol can be accepted for publication. You may also include additional comments for the author, including concerns about research or publication ethics.

You may also provide optional suggestions and comments to authors that they might find helpful in planning their study.

(Please upload your review as an attachment if it exceeds 20,000 characters)

Reviewer #1: This manuscript is essentially a study protocol to conduct a single-center, randomized controlled trial (RCT) for comparing the impact of Virtual reality (VR) immersion vs controls on IV sedation in adults undergoing breast surgery. The study was registered in clinicaltrials.gov, with a valid NCT number, and approved by the respective Ethics/IRB board. While the objectives and timeliness of this project appear sound and convincing, some comments appear below:

(a) The sample size/power stab doesn't mention the kind of statistical test used, whether the alternative hypothesis was one-or two-sided, and the desired effect size under consideration.

Thank you for your comment. The desired effect size was briefly mentioned on page 8:

"For our sample size calculation, we estimated a 30% reduction of propofol usage in the VR group compared to the control group."

We have now provided more information regarding our planned statistical analysis:

Added section :

"A two-sample t-test was used for the calculation, with a two-sided hypothesis test and a significance level of 0.05. To achieve 80% power, 90 participants were required, with the sample size inflated to 100 to account for attrition (e.g., dropout, protocol deviations)."

(b) Randomization: Details on the randomization is missing. Will it be a 1:1 randomization? What techniques will be used? Like, block randomization? If block, then what's the block size?

Thank you for this comment and we apologize for the oversight. This section has been clarified as such:

"Electronic randomization of the participants will be performed by an independent third party, using the National Cancer Institute's Clinical Trial Randomization tool. Specifically, we will randomize participants in a 1:1 ratio using a maximal randomization method, allowing a maximum tolerated imbalance of 3."

(c) Randomization: How are the blinding and allocation concealment (two separate steps) to be conducted? Again, details missing. To my surprise, this was mentioned as a limitation much later in the manuscript, whereas, the details regarding these should have appeared earlier.

Thank you for your comment. Our manuscript already notes that blinding of participants and personnel is not possible due to the nature of the virtual reality (VR) intervention. We recognize that a sham intervention, such as having a patient wear a non-functional VR headset, could theoretically be considered to achieve blinding. However, we believe this approach presents several important limitations:

1. A non-functional VR headset could potentially induce additional anxiety or feelings of claustrophobia, as it would restrict the participant's vision without providing the immersive, open 3D environment intended to reduce anxiety.
2. While a sham headset might initially mask group allocation from personnel, it would not effectively blind participants, who would likely realize they are not receiving an active VR

experience. This creates a significant risk that participants could inadvertently reveal their allocation during the procedure.

3. Importantly, our trial is designed to compare the VR intervention to the current standard of care. A sham headset does not represent standard practice and would therefore lack clinical relevance in this context.

We have added a passage in the discussion section regarding this comment.

As for the concealment method, it has also been mentioned in the assignment of interventions section:

"Each participant's allocation will be sealed in an opaque envelope and handed to the dedicated research staff not involved in patient care. The envelope will be opened at the patient's entry into the operating ward, after confirming that the surgery will be performed under regional anesthesia. Due to the nature of VR, the intervention cannot be blinded to participants or personnel after allocation."

Therefore, no changes were made in regards to this comment.

(d) Statistical Analysis: Please replace "multivariate" logistic regression (LR) with "multivariable" logistic regression.
Also, there is no mention of associated goodness-of-fit (GOF) assessments after conducting LR fits, say via Hosmer-Lemeshow GOFs.

Thank you for your comment.

"Multivariate" has been replaced by multivariable.

As for the second comment, we have also added a clarifying passage in the manuscript:

"To evaluate the fit of our logistic regression models, we will conduct the Hosmer-Lemeshow goodness-of-fit test. A p-value greater than 0.05 will be considered indicative of an adequate model fit. If poor fit is detected, alternative modeling strategies, such as transformations or interaction terms, will be considered."

(e) Conclusions/Discussion: A well thought out Conclusions/Discussion section is missing. In there, it should be mentioned that the expected results from this study is only for this sample of patients recruited at a Montreal hospital, and future trials with larger sample sizes and at other geographical locations will be necessary to assess the effectiveness of the VR.

Thank you for your comment.

The discussion section has been expanded to be more thoughtful and complete.

Reviewer #2: Thanks for the opportunity to review your manuscript/protocol. See below my major and minor comments

Major comments

1. What is the reason for not first performing a pilot/feasibility study instead immediately proceding to a randomized study. This rationale should be explicitly mentioned and in I would strongly urge you to reconsider, as a pilot study will provide pivotal information essential to more properly perform your randomized study.

Moreover, please provide evidence (or pilot data) that a VR intervention in this population is safe, and does not pose a higher risk for side effects like cybersickness. Finally, add data on the level of immersiveness of your specific VR intervention

Thank you for your comment.

We did not consider a pilot or feasibility study necessary in this case, as our trial builds upon an established body of literature with several similar trials already conducted using comparable methodologies. Many studies have shown that VR technology is relatively easy to implement in procedural contexts, and have also shown either equivalent or superior patient satisfaction in the VR group (https://pubmed.ncbi.nlm.nih.gov/38413184/) (https://pubmed.ncbi.nlm.nih.gov/28598921/) (https://pubmed.ncbi.nlm.nih.gov/36129891/) (https://pubmed.ncbi.nlm.nih.gov/32092098/) (https://pubmed.ncbi.nlm.nih.gov/38851457/). Also, we have recently performed a systematic review on intraoperative VR interventions and sedation usage reduction, which yielded promising results in this field (https://pubmed.ncbi.nlm.nih.gov/39862969/). While there are not yet any such studies specifically evaluating breast surgery patients under regional anesthesia, we have previously demonstrated that mastectomies performed under paravertebral block without general anesthesia are a safe and effective anesthetic strategy, providing benefits such as higher turnover rates, reduced postoperative nausea, and faster hospital discharge (https://pubmed.ncbi.nlm.nih.gov/34997554/). At our institution, this approach is now standard practice for mastectomy procedures. Additionally, prior to developing this trial, we informally tested the combination of paravertebral blocks with VR immersion in a small number of patients undergoing breast surgery under regional anesthesia, who tolerated the intervention well and reported high satisfaction. Given these factors, we believe that a pilot study is unnecessary.

Furthermore, patients undergo thorough screening to exclude those with conditions that could be exacerbated by VR usage, such as epilepsy. Cybersickness, a form of nausea induced by VR immersion, is a relatively well-known and anticipated side effect of this technology. However, previous trials have shown that it is infrequent and typically mild, with symptoms that are easily managed (https://pubmed.ncbi.nlm.nih.gov/28598921/, https://pubmed.ncbi.nlm.nih.gov/32092098/, https://pubmed.ncbi.nlm.nih.gov/32597390/).

A brief passage on cybersickness has been added to the discussion.

Finally, the level of immersiveness of the VR intervention will be addressed in the answer to your third comment.

2. The control group

As the intervention is aimed as a distraction method, it is likely more informative to include a different (less costly) distraction strategy as comparator. Please clarify the selection of your control group.

Thank you for your comment.

We acknowledge that comparing VR immersion to an alternative, less costly distraction strategy (e.g., music, guided imagery, or visual distraction) could offer valuable insights. However, we chose the current control group (no VR, standard care) based on two key considerations:

1. We recently conducted a systematic review of intraoperative VR interventions (https://pubmed.ncbi.nlm.nih.gov/39862969/). In all the trials included in this review, the control group consisted of standard care without any additional distraction techniques. Aligning our methodology with this established approach allows our trial to contribute meaningfully to the existing body of evidence and facilitate its inclusion in future meta-analyses.
2. Our primary objective is to evaluate whether VR immersion is beneficial as an adjunct to standard intraoperative care, rather than comparing it to another distraction method. Introducing a separate distraction intervention as a comparator would answer a different research question, namely, whether VR is superior to other distraction strategies. While this remains an important question, our current study aims to first establish the potential benefit of VR compared to standard practice in this specific surgical context. Future trials may build upon our findings to compare VR to other non-pharmacological interventions.

We hope this clarifies our rationale for the choice of control group and shows that this decision was made with careful consideration of both the existing evidence and our specific research objectives.

A passage relating to our rationale has been added to the discussion section.

3. The intervention group
Is the intervention VR-application fully immersive and includes a headphone. See for example https://pubmed.ncbi.nlm.nih.gov/34079940/ ; please explicitly describe whether this an commercial product or not
The possibility of different VR scenarios is nice, but add a level of variability that will hamper the effect of your intervention ans may result in treatment variation bias: please clarify (reconsider to perform a pilot study to select your most promising scenario)
It is unclear who accompanied patients during the VR intervention, as this maybe particularly relevant to the SoC and intervention effect on the primary endpoint (please take future implementation barriers into consideration)

Thank you for your comment.

We have clarified the device being used and the level of immersiveness in the interventions section.

“The VR headset to be used is the commercially available Paperplane Therapeutics VR system, specifically designed for medical applications. It offers a fully immersive experience incorporating both visual and auditory elements; notably, it is equipped with integrated near-ear speakers positioned along the side branches of the headset. These speakers are designed to direct sound toward the patient’s ears,

providing clear audio at a volume that is perceptible to the patient but remains low enough to avoid disturbing the surgical team or others in the operating room. This design also ensures that patients can continue to communicate freely with clinicians throughout the procedure."

Concerning the possibility of different VR scenarios, we understand the concern regarding the potential for increased variability and treatment variation bias. However, the inclusion of different scenarios is intentional and aligns with one of our secondary objectives, which is to explore whether passive (non-interactive) or interactive VR experiences are more effective in reducing intraoperative medication requirements. Indeed, prior literature suggests that interactive VR content may have superior anxiolytic properties compared to passive experiences, but this has only been established in the context of dental surgery (http://pubmed.ncbi.nlm.nih.gov/37993645/). Therefore, we found it interesting not only to assess the overall efficacy of VR but also to provide preliminary insights into the impact of scenario interactivity on sedation needs.

While this approach introduces some degree of heterogeneity, we aim to document each participant's scenario selection and account for this variability in our subgroup analyses, which we believe will mitigate the risk of treatment variation bias. Just as not all movies appeal to all audiences, our study explores a real-world application of VR where patients have autonomy in selecting their VR scenario.

Finally, concerning the person who accompanies patients during the VR intervention, we have added a passage in the interventions section explaining who will accompany the patient:

"A dedicated research staff member will be present in the operating room throughout the procedure to manage all aspects of the VR intervention, including assisting with headset application, adjusting the VR scenarios upon patient requests, and addressing any technical issues or patient discomfort related to the VR system."

We have also added a passage in the discussion relating to future implementation.

"Although dedicated research staff will be responsible for managing all aspects of the VR intervention during our trial, we recognize that successful integration of this technology into routine clinical practice will ultimately require training regular hospital personnel in the application and operation of the device. This could involve orderlies, nurses, or even attending anesthesiologists, ensuring that VR-assisted anxiolysis can be seamlessly incorporated into standard intraoperative workflows."

4. Outcomes

Please provide clarity on how you operationalization your secondary and exploratory endpoints as these are currently partly missing in the manuscript (lines 159-178)

Thank you for your comment.

We have added information where required and clarified the operationalization for each individual outcome (see **2.1.4. Outcomes** in the manuscript).

The background of the Nociception Level (NOL) index is poorly mentioned, and as such the rationale to include NOL as exploratory outcome is unclear. Please elaborate

A background paragraph on the NOL index with a rationale for the exploration of its use has been added to the introduction, prior to the objectives.

[...] Not including longer-term (patient-centered) outcomes is a missed opportunity (see also L330-331). Please explain why you did not include such relevant outcome (and reconsider)

We recognize the importance of long-term, patient-centered outcomes such as postoperative pain, functional recovery, and patient satisfaction beyond the immediate perioperative period. However, the primary objective of this trial is to evaluate the intraoperative effects of VR immersion on sedation requirements and patient anxiety during surgery under regional anesthesia. As such, our focus is on the immediate intraoperative and early postoperative (PACU) period, where the impact of VR on sedation and acute recovery is most directly observable. We felt that this was most relevant to the immediate goals of VR as a non-pharmacological anxiolytic and sedation-sparing tool in the intraoperative setting.

5. Sample Size

Please elaborate and provide references that substantiate that a 30% reduction in clinically relevant. In addition, provide historical data on propofol requirements during the surgical procedure under study.

Thank you for your comment.

Available evidence suggests that dose reductions in this range can lead to meaningful clinical improvements and have been considered a clinically significant benefit to patients based on previous research. For instance, in a study comparing patient-controlled sedation (PCS) to clinician-controlled sedation found a 39.3% reduction in propofol usage in the PCS group, which correlated with fewer episodes of deep sedation, fewer airway and breathing adverse events, and faster recovery times (https://pmc.ncbi.nlm.nih.gov/articles/PMC8787772/). Similarly, in a study evaluating lidocaine as an adjunct to reduce propofol requirements during pediatric colonoscopy, the lidocaine group consumed 35.5% less propofol than the control group, which was associated with significantly shorter awakening times ($P < 0.001$), faster recovery times ($P < 0.001$), and fewer involuntary movements during the procedure ($P = 0.028$) (https://pubmed.ncbi.nlm.nih.gov/34852767/). Therefore, we believe that a 30% reduction in propofol usage would similarly represent a clinically significant improvement in our trial population.

Furthermore, paravertebral block with propofol sedation has been validated as a viable alternative to general anesthesia for breast cancer surgery, offering potential benefits such as higher turnover rates, reduced postoperative nausea, and faster hospital discharge (https://pubmed.ncbi.nlm.nih.gov/34997554/). At our institution, this approach is now standard practice for mastectomy procedures. Concerning historical propofol requirements for the surgical procedure in question, in the study by Sessler et al., patients in the paravertebral block group received a median propofol dose of 525 mg per patient (https://pubmed.ncbi.nlm.nih.gov/31645288/).

A passage detailing some of this information has been added to the “Sample size” section.

6. Consider including the recently published ‘Reporting Guidelines for the Early-Phase Clinical

Evaluation of Applications Using Extended Reality: RATE-XR Qualitative Study Guideline’ (PMID: 39612482) and add its items that are currently missing into your manuscript.

Thank you very much for drawing our attention to these reporting guidelines.

While we understand that the checklist is primarily intended for reporting the results of completed trials, we have made every effort to incorporate the relevant elements into our protocol as suggested. The updated manuscript now comprises all sections from these guidelines. We mainly added sections relating to the VR device itself, added justifications for our sample size and discussed future implementation considerations, among other minor modifications.

Minor comments
L34: 'explore' change into 'determine'

This has been done.

L36 include description of the application used in your abstract

A brief description has been added.

L78-80: add reference to substantiate your claim; moreover, add a sentence on standard anesthesia during this surgical procedure

A consolidated passage on this has been added.

L85: appreciate change into determine

This has been done.

L104: omit minimal risk (see also L267). Add single-center 1. minimal risk is not part of study design; 2. this is your assumption: please elaborate your arguments that this is a low-risk study

It has been omitted.

L115: add age cut-off for your adult patients

This has been added.

L120 Eligibility criteria: Severe mental impairment: How operationalized?

Thank you for this comment. We acknowledge the lack of clarity in this eligibility criterion. What we meant was “Cognitive or psychiatric impairment that would interfere with informed consent, patient-controlled sedation use, or VR engagement”.

To ensure objectivity, this criterion will be assessed based on a documented clinical diagnosis in the patient's medical record. If there is significant clinical doubt regarding a patient's cognitive abilities, a Montreal Cognitive Assessment (MoCA) test will be administered by our trained research nurse, who is qualified to conduct cognitive assessments, as done in previous research projects on postoperative cognitive impairment.

This has been changed appropriately.

L153-154: please provide your pre-specified criteria for switching to general anesthesia

We have provided the pre-specified criteria.

L159-160: Validated method?

Thank you for your inquiry. All preoperative and postoperative questionnaires were screened and validated by our institution's ethical committee.

L161: please add the rationale to collect data on pre-operative anxiety, and how you measure this (and its cut-off)

Thank you for your comment. The rationale for assessing preoperative anxiety is to evaluate whether it could act as a potential confounder, as randomization may not always achieve perfect balance between groups. This has been added to the objectives section.

There is no pre-specified cut-off for anxiety levels, as anxiety level is not an exclusion factor. Preoperative anxiety and information requirements will be assessed using the validated Amsterdam Preoperative Anxiety and Information Scale (APAIS), a six-item questionnaire designed to measure both anxiety and the desire for information before surgery. The scale provides an overall anxiety score ranging from 4 to 20 and an information-seeking score ranging from 2 to 10, with higher scores indicating greater preoperative anxiety and a stronger desire for information, respectively.

This has been added to section **2.3.1. Data collection methods** in the manuscript.

L187 please specify 'variety'

Thank you for your comment. For the sake of conciseness, we utilized the term variety, as all assessments were outlined appropriately in Fig. 2 (the Schedule of enrolment, interventions and assessments).

L217-218 please clarify the questionnaire used (reference, validated? Self-composed?)

Thank you for your comment. All of our questionnaires were screened and validated by our institution's ethical committee. In this case, the preoperative questionnaire that was mentioned was self-composed by us and linked in the supplementary materials, as were all other questionnaires.

We have added "custom-designed questionnaire" in the description.

L254-255: please clarify and specify: 'if relevant', 'additional variables' what is the outcome of interest?

Thank you for your comment.

We have clarified the outcome of interest (total propofol consumption) and have better clarified in which situations multivariable regression will be used:

"If preliminary analyses indicate significant associations ($P < 0.05$ in univariate analysis), multivariable analyses will be conducted to assess the combined influence of key independent variables (e.g. preoperative anxiety levels, age, BMI, etc.)."

L255-257: please clarify, and omit secondary. Which subgroups?

Thank you for your comment, the sentence has been clarified:

"Subgroup analysis will also be performed to explore how the selected initial VR scenario may impact our primary outcome."

It is now clear the subgroup analyses will be conducted based on the initial VR scenario chosen.

L257-258 please clarify what you intend to do?

Thank you for your comment, this has been clarified:

"In the case of conversion to general anesthesia, the participant will be considered a protocol deviation. These participants' data will still be included in the final analysis following an intention-to-treat (ITT) approach. Additionally, we will compare the prevalence of protocol deviations between the intervention and control groups to assess whether the conversion rate differs between conditions."

L263: please provide the level of statistical significance

The level of statistical significance has been provided:

"[...] statistical significance will be determined using an alpha level of 0.05."

L275-276: please specify how your screen for, define your side effects (see also L162-164)

Thank you for these comments.

We have specified our screening process:

“Participants at higher risk of serious side effects (e.g. seizures) will be identified through medical chart review and self-reported medical history and excluded from the study during the screening process.”

The side effects have been defined:

“Incidence of intraoperative and postoperative adverse events such as bradycardia (heart rate < 50 bpm), desaturation (SpO2 < 92%) and hypotension (MAP < 60) as well as subjective adverse events, such as cybersickness or nausea documented intraoperatively by the anesthesiologist and research staff based on real-time clinical observation or patient self-reporting”

L276-277: omit, as already mentioned in the exclusion criteria

This has been omitted.

Reviewer #3: Thank you for the opportunity to review this protocol manuscript, “Virtual reality as a strategy for intra-operatory anxiolysis and pharmacological sparing in patients undergoing breast surgeries: the V-RAPS randomized controlled trial.” The study is well designed and the protocol follows the SPIRIT guidelines. I have a few points for consideration by the authors.

L 103: Was this definition of minimal risk one used by your IRB or is this your opinion? Often randomized trials that involve drugs, especially self administered sedatives, are considered “full board” and not minimal risk.

Thank you for the comment. The term “minimal risk” referred to the VR equipment usage rather than the self-administered propofol, and in both cases, this was not officially used by our IRB. As such, we have changed our phrasing appropriately.

In the action scenario, the patients are allowed to move their hands and arms freely?

Thank you for this comment. The action scenarios rely solely on eye-tracking software for the direction of the “in-game” cursor. As such, there is no necessity for excessive arm and hand movement other than simply pressing the trigger buttons on the controller. This has been specified in the manuscript:

“Notably, the interactive VR scenarios do not require significant hand or arm movement, as they rely solely on eye-tracking software to aim within the game. The only manual action required is pressing the controller trigger at specific time points.”

L163: Define hypotension and hypoxia

Thank you for this comment. These have been defined accordingly.

L175: Explain why you would not assess satisfaction in the control group. Wouldn’t patient satisfaction with their anesthesia experience be worth comparing?

Thank you for your comment. You are right, this is definitely something that would be worth comparing. We have added this outcome to the manuscript.

L257: If the patient requires general anesthesia, is this recorded separately as a secondary outcome? Is the primary outcome data still analyzed?

Thank you for your comment. We apologize for the initial lack of clarity, we have better specified our methods in the case of a protocol deviation:

“In the case of conversion to general anesthesia, the participant will be considered a protocol deviation. These participants' data will still be included in the final analysis following an intention-to-treat (ITT) approach. Additionally, we will compare the prevalence of protocol deviations between the intervention

and control groups to assess whether the conversion rate differs between conditions. To minimize protocol deviations, patient recruitment and randomization will only be finalized after the paravertebral block has been performed and its effectiveness has been confirmed by the lead clinician. If the block is deemed grossly ineffective, the patient will not be recruited or randomized."

L261: Provide more details on how you will make a predictive model for NOL spikes and subsequent propofol bolus

Thank you for your comment.

We have clarified this in the methodology.

"As part of our exploratory analysis, we will investigate the relationship between the NOL index and propofol self-administration to determine whether NOL spikes serve as a predictor of subsequent propofol boluses. To achieve this, we will define a spike as any NOL score exceeding a threshold of 50 for a pre-specified duration of at least 10 seconds. We will then use time-series analysis to assess the temporal relationship between NOL spikes and propofol bolus administration. This will allow us to apply logistic regression and/or time-to-event analysis to determine whether NOL spikes significantly predict the likelihood of a propofol bolus within a predefined timeframe of up to 60 seconds."

L271: Any potential harms associated with patient self administered propofol?

Thank you for your comment.

Interestingly enough, patient-controlled sedation with propofol has been shown to be equivalent, if not safer than clinician-controlled sedation (https://pubmed.ncbi.nlm.nih.gov/29750696/). It has also been shown to reduce sedation requirements (https://pubmed.ncbi.nlm.nih.gov/34852928/).

Furthermore, it is always done under anesthesiologist supervision and has a pre-specified lockout period during which the patient cannot receive sedative medication.

L321: It is possible, however difficult, to blind anesthesia and surgery personnel to the study assignment by using sham equipment and limiting verbal communication about the experience. If you chose not to use blinding in your study, I would recommend changing the phrasing from "it is not possible" to "we decided" etc. An additional limitation is that your study does not control for the placebo effect, which also could have been accounted for by the use of sham equipment.

Thank you for your comment.

We have dedicated a larger discussion section to this topic.

"Due to the nature of VR interventions, it is difficult for the patient, the anesthesiologist or the surgeon to be blinded to group allocation. We recognize that a sham intervention, such as having a patient wear a non-functional VR headset, could theoretically be considered to achieve blinding. However, we believe this approach presents several important limitations.

Firstly, a non-functional VR headset could potentially induce additional anxiety or feelings of claustrophobia, as it would restrict the participant's vision without providing the immersive, open 3D environment intended to reduce anxiety; This unintended discomfort could, in turn, bias the results of our trial by exaggerating the perceived benefit of the active VR intervention. Secondly, while a sham headset might initially mask group allocation from personnel, it would not effectively blind participants, who would likely realize they are not receiving an active VR experience. This creates a significant risk that participants could inadvertently reveal their allocation during the procedure, which would hamper the effects of the blinding. Lastly, our trial is designed to compare a VR intervention to the current standard of care; a sham headset does not represent standard practice and would therefore lack clinical relevance in this context.

While our trial will not utilize blinding, the use of PCS minimizes the anesthesiologist's influence on propofol administration, reducing performance bias."

L338: VR headsets and software cost money. Would you not consider Paper Plane's provision of these materials to be industry support and worth mentioning in the funding section?

Thank you for your comment.

This has been clarified as follows in the online submission for and has been removed from the manuscript, as per the editor's recommendations :

"Funding: This study will be funded by Dr. Pascal Laferrière-Langlois from CR-HMR and the Department of Anesthesiology and Pain Medicine. The hardware and software will be provided free of charge by Paperplane Therapeutics. No funding was sought for the redaction of this protocol."

7. PLOS authors have the option to publish the peer review history of their article (what does this mean?). If published, this will include your full peer review and any attached files.

If you choose “no”, your identity will remain anonymous but your review may still be made public.

Do you want your identity to be public for this peer review? For information about this choice, including consent withdrawal, please see our Privacy Policy.

Reviewer #1: No

Reviewer #2: **Yes:** Evert-Jan Wils

Reviewer #3: No
