# Supplementary material for: Virtual reality as a strategy for intra-operatory anxiolysis and pharmacological sparing in patients undergoing breast surgeries: The V-RAPS randomized controlled trial protocol
Source: PLoS One. 2025 Jul 7;20(7):e0327555. doi: 10.1371/journal.pone.0327555 (PMC12233254; doi:10.1371/journal.pone.0327555)
Supplement: Attachment — Submitted filename: VRAPS_Review2.pdf [file pone.0327555.s006.pdf]

Comments to the Author

1. Does the manuscript provide a valid rationale for the proposed study, with clearly identified and justified research questions?

The research question outlined is expected to address a valid academic problem or topic and contribute to the base of knowledge in the field.

Reviewer #1: Yes

Reviewer #2: Yes

2. Is the protocol technically sound and planned in a manner that will lead to a meaningful outcome and allow testing the stated hypotheses?

The manuscript should describe the methods in sufficient detail to prevent undisclosed flexibility in the experimental procedure or analysis pipeline, including sufficient outcome-neutral conditions (e.g. necessary controls, absence of floor or ceiling effects) to test the proposed hypotheses and a statistical power analysis where applicable. As there may be aspects of the methodology and analysis which can only be refined once the work is undertaken, authors should outline potential assumptions and explicitly describe what aspects of the proposed analyses, if any, are exploratory.

Reviewer #1: Yes

Reviewer #2: Yes

3. Is the methodology feasible and described in sufficient detail to allow the work to be replicable?

Descriptions of methods and materials in the protocol should be reported in sufficient detail for another researcher to reproduce all experiments and analyses. The protocol should describe the appropriate controls, sample size calculations, and replication needed to ensure that the data are robust and reproducible.

Reviewer #1: Yes

Reviewer #2: Yes

4. Have the authors described where all data underlying the findings will be made available when the study is complete?

The PLOS Data policy requires authors to make all data underlying the findings described in their manuscript fully available without restriction, with rare exception, at the time of publication. The data should be provided as part of the manuscript or its supporting information, or deposited to a public repository. For example, in addition to summary statistics, the data points behind means, medians and

variance measures should be available. If there are restrictions on publicly sharing data—e.g. participant privacy or use of data from a third party—those must be specified.

Reviewer #1: Yes

Reviewer #2: Yes

5. Is the manuscript presented in an intelligible fashion and written in standard English?

PLOS ONE does not copyedit accepted manuscripts, so the language in submitted articles must be clear, correct, and unambiguous. Any typographical or grammatical errors should be corrected at revision, so please note any specific errors here.

Reviewer #1: Yes

Reviewer #2: Yes

6. Review Comments to the Author

Please use the space provided to explain your answers to the questions above and, if applicable, provide comments about issues authors must address before this protocol can be accepted for publication. You may also include additional comments for the author, including concerns about research or publication ethics.

You may also provide optional suggestions and comments to authors that they might find helpful in planning their study.

(Please upload your review as an attachment if it exceeds 20,000 characters)

Reviewer #1: The authors were able to address my previous comments with a great degree of satisfaction. I have no further comments.

We sincerely thank you for the time and energy you have invested into the betterment of our manuscript.

Reviewer #2: Thanks for the opportunity to review your revised manuscript/protocol. Your adjustments and answers have added clarity to the study design and the rationale of your choices made.

Thank you very much for your comments and dedication to improving our manuscript; it is very much appreciated.

I have only a few minor follow-up comments:

1. As your study compares the VR-intervention with standard of care (control rather than other method), your study is pragmatic by nature. It will not be able to differentiate between an effect of the interface or the content (despite the non-randomized choice for scenario by patients). Consider to add pragmatic into the phrasing of the relevant paragraph (L417-428)

We thank you for this valuable comment and agree with your assessment. This phrasing has been added accordingly in the relevant paragraph:

"Furthermore, our primary objective is to evaluate whether VR immersion is beneficial as an adjunct to standard intraoperative care, rather than comparing it to another distraction method, which would answer a different research question. While this is also an interesting question, our current study aims to first establish the potential benefit of VR compared to standard practice in this specific surgical context. **As such, the design of our study is pragmatic in nature, focusing on real-world implementation of VR technology in operative settings, including non-randomized scenario selection**. Future trials may build upon our findings to compare VR to other non-pharmacological interventions **or to formally evaluate differences between VR scenario types.**"

2. Although the secondary objective on (non-interactive) or interactive VR is of interest, I would remain hesitant in general to make adjustments in your study design to facilitate your secondary objective that may hamper robustness of your primary objective (previous major comment 3). Moreover, to account for this variability in intended subgroup analysis remains cumbersome, as it is non-randomized. Please add this into your limitation section.

Thank you once more for your attention to our secondary objective. You are correct in stating that the results of these subgroup analyses should be interpreted carefully. To reflect this, we have added an additional limitation in our discussion:

"One of this study's secondary objectives is the analysis of subgroup outcomes based on the choice of initial VR scenario; specifically, it aims to assess whether interactive or non-interactive scenarios may have a different effect on sedation requirements. However, as previously mentioned, scenario choices will not be randomized, but rather chosen based on participant preference, as this is a pragmatic trial with the goal of eventual real-world implementation. While this grants participants an opportunity to partake in a personalized experience, it may create a form of selection bias in our subgroup analyses. Therefore, we acknowledge that any results arising from such analyses should simply be considered as hypothesis-generating rather than robust evidence, and will be framed as such."

3. L217-218 please clarify the questionnaire used (reference, validated? Self-composed?). The fact that your self-reported preoperative questionnaire has been screened and validated by your institution's ethical committee, does not imply that the questionnaires are validated scientifically. Please rephrase custom-designed questionnaire into self-composed, and add a sentence into the manuscript that it is not (yet) validated.

Thank you for your comment. We agree that including such information will better contribute to our transparency. For the sake of conciseness, we have chosen to include this information in the "Data collection methods" section of the methodology rather than on L217-218 (in the objectives section), as this would require us to repeat the information for every item that is assessed on a questionnaire, making that section lengthier than it should be.

"We will collect data at baseline, intraoperatively and postoperatively to assess primary and secondary outcomes. Participant socio-demographic information, medical history and current medication, will be collected from their medical chart. Their education level and familiarity with VR technology will also be recorded through a preoperative questionnaire. Pre-operative anxiety levels will be assessed using the APAIS, a six-item questionnaire designed to measure both anxiety and the desire for information before surgery. The scale provides an overall anxiety score ranging from 4 to 20 and an information-seeking score ranging from 2 to 10, with higher scores indicating greater preoperative anxiety and a stronger desire for information, respectively. A postoperative questionnaire will be used to collect data on patient satisfaction. **It is of note that, aside from the APAIS, both our preoperative and postoperative questionnaires are self-composed and have not yet undergone formal scientific validation.**"

Furthermore, we have added a reference for the first time the Amsterdam Preoperative Anxiety and Information Score (APAIS) was mentioned, in the objectives section.

7. PLOS authors have the option to publish the peer review history of their article (what does this mean?). If published, this will include your full peer review and any attached files.

If you choose “no”, your identity will remain anonymous but your review may still be made public.

Do you want your identity to be public for this peer review? For information about this choice, including consent withdrawal, please see our Privacy Policy.

Reviewer #1: No

Reviewer #2: Yes: Evert-Jan Wils
